# Organoids and Colorectal Cancer

**DOI:** 10.3390/cancers13112657

**Published:** 2021-05-28

**Authors:** Antonio Barbáchano, Asunción Fernández-Barral, Pilar Bustamante-Madrid, Isabel Prieto, Nuria Rodríguez-Salas, María Jesús Larriba, Alberto Muñoz

**Affiliations:** 1 Instituto de Investigaciones Biomédicas “Alberto Sols”, Consejo Superior de Investigaciones Científicas (CSIC), Universidad Autónoma de Madrid (UAM), 28029 Madrid, Spain; abarbachano@iib.uam.es (A.B.); afbarral@iib.uam.es (A.F.-B.); pbustamante@iib.uam.es (P.B.-M.); mjlarriba@iib.uam.es (M.J.L.); 2 Centro de Investigación Biomédica en Red-Cáncer (CIBERONC), 28029 Madrid, Spain; nuria.rodriguez@salud.madrid.org; 3 Instituto de Investigación Sanitaria del Hospital Universitario La Paz (IdiPAZ), 28046 Madrid, Spain; 4 Servicio de Cirugía General, Hospital Universitario La Paz, 28046 Madrid, Spain; iprieto@intermic.com; 5 Servicio de Oncología Médica, Hospital Universitario La Paz, 28046 Madrid, Spain

**Keywords:** organoids, patient-derived organoid/PDO, patient-derived tumor organoid/PDTO, colon, colorectal, cancer

## Abstract

**Simple Summary:**

Colorectal cancer is one of the most frequent and lethal types of cancer. Despite advances in recent decades, our knowledge of this disease is still limited, and novel and better therapies are needed. Organoids were recently developed as a new system to culture normal and tumor cells obtained from patients subjected to surgery or endoscopic tests. Organoids are being used to dissect the molecular and genetic bases of colorectal cancer initiation and progression. In this review, we describe how patient-derived organoids can be generated, and their use to investigate in depth the tumorigenic process. We show how this system has allowed the study of colorectal tumorigenesis features for the first time, including immunotherapy, interplay with microorganisms and, more importantly, assays of drug treatments at an individualized level. Additionally, we summarize the most recent developments of what is known as organoid technology directed towards personalized medicine.

**Abstract:**

Organoids were first established as a three-dimensional cell culture system from mouse small intestine. Subsequent development has made organoids a key system to study many human physiological and pathological processes that affect a variety of tissues and organs. In particular, organoids are becoming very useful tools to dissect colorectal cancer (CRC) by allowing the circumvention of classical problems and limitations, such as the impossibility of long-term culture of normal intestinal epithelial cells and the lack of good animal models for CRC. In this review, we describe the features and current knowledge of intestinal organoids and how they are largely contributing to our better understanding of intestinal cell biology and CRC genetics. Moreover, recent data show that organoids are appropriate systems for antitumoral drug testing and for the personalized treatment of CRC patients.

## 1. Introduction

### 1.1. Organoid Definition

Organoid technology is revolutionizing research in biology and biomedicine with an increasing clinical impact on personalized medicine. A decade after its emergence, the number of published studies using this technology is growing exponentially, and several excellent reviews have recently summarized the available data, technical advances and results obtained in a large variety of biological systems and human diseases [1,2,3,4]. Here, we will focus on the impact that organoids are having on our knowledge and perspectives of colorectal cancer (CRC), one of the main neoplasias in terms of incidence and mortality worldwide.

**Figure 1:**
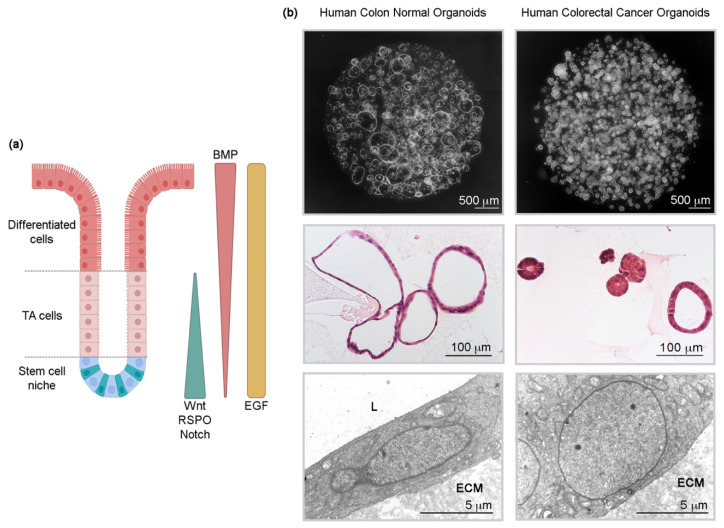
(**a**) Scheme of a colon crypt including some of the niche factors controlling cell fate. TA cells, transient-amplifying cells. Created with Biorender.com (accessed on 27 April 2021). (**b**) Morphology of human colon normal (PDO) and tumor (PDTO) organoids. Upper panels: light microscopy images. Middle panels: hematoxylin and eosin (H&E) staining. Lower panels: electron microscopy images showing the common undifferentiated phenotype of cells in both types of organoids (decondensed euchromatin, lack of villi, scarce cytoplasmic organella). ECM, extracellular matrix (Matrigel); L, lumen. Images shown are unpublished and correspond to studies by our group.

Definitions of the term organoid have been provided by several key researchers in the field [3,5,6]. As expected, the definitions are similar and basically describe organoids as three-dimensional (3D), self-organized multicellular structures generated by stem cells (SC: embryonic (ESC), adult (ASC) or induced pluripotent (iPSC)) or cancer stem cells (CSC) embedded in an extracellular matrix (ECM) (Figure 1). They have long-term proliferation and differentiation capacities, depending on the culture medium, and recapitulate some of the features of a particular organ or tumor of origin. As discussed in the following sections, patient-derived organoids (PDOs) or patient-derived tumor organoids (PDTOs) reproduce the in vivo tissue situation much better than the immortalized cell lines grown on 2D plastic dishes for decades.

Notably, non-stem or stem cell aggregates growing in suspension (spheroids) and organoids established from patient-derived tumors transplanted in mice should be distinguished from PDOs that are directly generated from human primary SC. Although some authors consider PDOs miniaturized organs, the nature of ASC-derived organoids is exclusively epithelial (SC and their variably differentiated daughter cells). By contrast, iPSC-derived organoids may contain epithelial and mesenchymal cells.

### 1.2. Colorectal Cancer (CRC)

CRC is a heterogenous group of neoplastic diseases that result from the malignant transformation of the epithelium lining the external surface of the large intestine. This transformation or change in cell phenotype occurs as a consequence of genetic alterations (mutations) and epigenetic modifications which are thought to progressively accumulate over 10–20 years [7,8], although abrupt episodes of synchronous multiple mutagenesis (chromothripsis, chromoplexy, polyploidization, *kataegis*) can also take place [9]. Molecular subtypes of CRC with partially different alterations, biological features and clinical behavior have been proposed. Perhaps the most widely accepted today is that of Guinney and colleagues [10,11]. These CRC subtypes distribute unevenly along colon segments and the rectum and contribute to differences in clinical development, management and response to therapies for right/ascending colon, left/descending colon and rectal tumors [12,13]. However, most CRC display alterations in proto-oncogenes and tumor suppressor genes, leading to the deregulation of a few signaling pathways: WNT/β-catenin, EGFR-RAS-RAF, PI3K, p53, and TGF-β-SMADs [7,8]. Unfortunately, the great improvement in the knowledge of CRC at molecular and genetic level in recent decades has not yet been translated into an equal increase in clinical benefit for patients. As in many other cancer types, one possible reason for this has been the lack of models that appropriately reproduce the in vivo situation and can successfully be used for disease modelling and drug testing. This represents an opportunity for PDOs, as discussed in the following sections. The CSC hypothesis states that the genetic/epigenetic alteration of tissue-resident SC is the origin of tumors and probably also of their progression and relapses on treatment. A key issue in this hypothesis has been the characterization of CSC in each type of tumor. This remained elusive for a long time in the case of CRC (and many other neoplasias). Another major unresolved point that has long hindered advances in the understanding and therapy of CRC has been the lack of ability to culture normal intestinal epithelial cells isolated from biopsies. These shortcomings have been solved successfully by organoid technology.

### 1.3. The Short History of Intestinal Organoids

The intestinal epithelium is the tissue with the most rapid self-renewal capacity in adult mammals. This cellular turnover is mediated by a group of SC. However, their location and specific marker genes were unknown for many years, making their study a difficult challenge. Monolayer-grown primary cultures obtained from normal intestinal tissue were short-lived because cells were mostly differentiated, and the standard culture medium was not appropriate for SC growth. This scenario changed radically with the discovery by the Clevers’ group of leucine-rich repeat-containing G-protein coupled receptor 5 (Lgr5), also known as G-protein coupled receptor 49 (Gpr49), as a SC marker in the mouse small intestine [14]. Lgr5 is a transmembrane-G-protein-coupled receptor that binds to its ligands, secreted R-spondins, to potentiate the Wnt/β-catenin pathway [15,16,17]. This finding allowed the Clevers’ group to locate SC in the intestinal crypt bottom, and subsequently made possible their isolation and 3D culture in vitro as organoids, first from normal mouse small intestine [18] and later on from normal human colon [19,20]. Lgr5 expression is not exclusive to the intestine. It is present in many body tissues, such as the stomach, lung, prostate, mammary gland, tongue, liver, pancreas and others [21]. This led to the identification of SC and the establishment of organoid cultures from many tissues.

The mutation of the *Adenomatous polyposis coli* (*Apc*) tumor suppressor gene is a key initial and common event in CRC tumorigenesis. Using an *Apc* knock-in mouse model, Barker and colleagues demonstrated that only *Apc* mutant *Lgr5^+^* cells can form adenomas. This suggests that mutated crypt SC are the origin of intestinal cancer. Furthermore, in lineage tracing studies, Schepers and colleagues showed that these adenomas maintain *Lgr5^+^* stem cell activity [22,23].

The success of establishing intestinal organoids was based on embedding either intestinal crypts or single normal or tumor cells in an ECM covered by a medium containing a complex cocktail of factors and inhibitors [18,19,20,24] (Table 1). These conditions reproduced the SC niche found at the intestinal crypt bottom in vivo and allowed SC to proliferate and differentiate into committed progenitor and more differentiated cells that self-organize in 3D structures with variable morphology and usually an internal lumen where the most differentiated apoptotic cells are expelled. In this way, these organoids allowed for the first time the long-term culture of normal and tumor intestinal SC from tissue biopsies without performing any type of genetic modification. Thus, organoids constitute a good model system to study intestinal homeostasis and epithelial cancers (carcinomas) such as CRC.

## 2. Normal and Tumor Intestinal Organoid Culture

The basic idea to culture intestinal SC in vitro is to use a medium that reproduces the conditions (a composition of factors that determine the cell fate) present at their niche. At the intestinal crypt bottom, the activation of WNT/β-catenin and Notch pathways together with the blockade of bone morphogenetic protein (BMP) signaling by secreted antagonists (Noggin, Gremlin, Chordin) are essential for cell stemness. Epidermal growth factor receptor (EGFR) ligands act as mitogens. WNT/β-catenin and Notch signals decrease upwards along the crypt, while BMP increases, resulting in cell differentiation [28] (Figure 1). Paneth cells, intercalated between intestinal SC, provide the crucial WNT and Notch signals in small intestine [29]. In the colon, where Paneth cells are absent, these signals are supplied by the stroma and the deep crypt secretory cells [30,31].

Culture medium for mouse normal colon organoids requires a basis of Advanced DMEM/F12/Glutamax/HEPES medium supplied with WNT signaling activators: R-spondin and conditioned medium from Wnt3A-expressing cells. The latter is preferred over recombinant WNT protein because of its post-translational modifications (fatty acids) and inherent instability due to its high hydrophobicity. Some studies have proposed the use of phospholipids and cholesterol as carriers or the glycoprotein afamin to improve WNT protein stability [32,33]. Additionally, the medium contains Noggin to inhibit BMP-induced differentiation [34], the mitogen epidermal growth factor (EGF) [35], and the Rho-associated protein kinase (ROCK) inhibitor Y-27632 to prevent *anoikis* (apoptosis due to lack of anchorage to substrate) [36,37]. Antibiotics are required to avoid bacterial contamination, and N-acetyl-L-cysteine and the N2 and B27 supplements containing mixtures of vitamins and cofactors are also commonly added (Table 1).

Normal human colon organoids also demand gastrin, A 83-01 or LY2157299 to inhibit the antiproliferative effect of TGF-β, and SB202190 to block the negative feed-back effect of p38 mitogen-activated protein kinase (p38MAPK) on EGFR. Although probably not strictly required, nicotinamide and prostaglandin E_2_ (PGE_2_) are used to maintain cell stemness and the cystic phenotype of normal organoids [19,20,38]. Remarkably, Sato’s group increased the efficiency of human organoid growth by replacing SB202190 with fibroblast growth factor (FGF)-2 and insulin-like growth factor (IGF)-1 [24]. Today, most studies refer to nearly 100% efficiency in normal PDO establishment. Curiously, the histone deacetylase inhibitor valproic acid (a Notch activator) and the glycogen synthase 3 inhibitor CHIR99021 (CHIR, a WNT/β-catenin activator) have been proposed to synergistically increase the proliferation of mouse intestinal *Lgr5^+^* cells [39]. However, they inhibit the proliferation of mouse cells with a mutated *Apc* gene, perhaps due to an excessive activation of WNT signaling [40]. CHIR has also been used to enhance the recovery of human intestinal single cells [25].

Importantly, organoid culture medium optimized for human normal colon tissue is suitable for the expansion of tumor colon tissue cells with minor modifications (Table 1). Over 94% of CRC display the mutually exclusive mutation of certain genes encoding key components of the WNT/β-catenin pathway *(APC*, *CTNNB1* or *AXIN)*, which makes it highly likely that CRC PDTOs have a constitutive activation of this pathway. This means that the use of Wnt3A-conditioned medium to culture CRC PDTOs is dispensable. Remarkably, the absence of WNT precludes the growth of nontumor cells in PDTO cultures. Nevertheless, the efficiency of CRC PDTO establishment usually reaches around 70%, which means that the medium is not yet optimized [41,42]. Sato’s group refined the culture medium by combining the presence or absence of Wnt3A, SB202190 and oxygen concentrations, which increased the efficiency of tumor organoid establishment up to 100%. They also reported the culture of organoids from rare colon neuroendocrine tumors [26,27]. The decrease in niche factor dependency due to the accumulation of alterations along the adenoma−carcinoma progression can make some factors of the culture medium dispensable for certain PDTOs [19,26,43,44,45].

For CRC PDOs and PDTOs (in fact for all epithelial PDOs), embedding into an ECM that recreates the 3D environment in vivo is a requisite. The standard ECM used is Matrigel or basement membrane extract (BME) purified from the Engelbreth-Holm-Swarm (EHS) mouse sarcoma, whose major components are laminin, collagen IV and entactin. Matrigel also contains a batch-dependent variable low amount of EGF, TGF-β, IGF-1 and basic FGF.

## 3. Organoids as a Model to Study CRC

Today, organoids are accepted as a better system to study cancer genetics, cancer processes and the activity of antitumor drugs than cell line monolayers and genetically engineered mouse model. Of note, the most commonly used animal model for CRC is the *Apc^min^* mouse that carries a germline mutated *Apc* allele and spontaneously develops adenomas early in life. These animals later develop many tumors in the small intestine, but very few in the colon, and so are at most a model for familial adenomatous polyposis.

**Figure 2:**
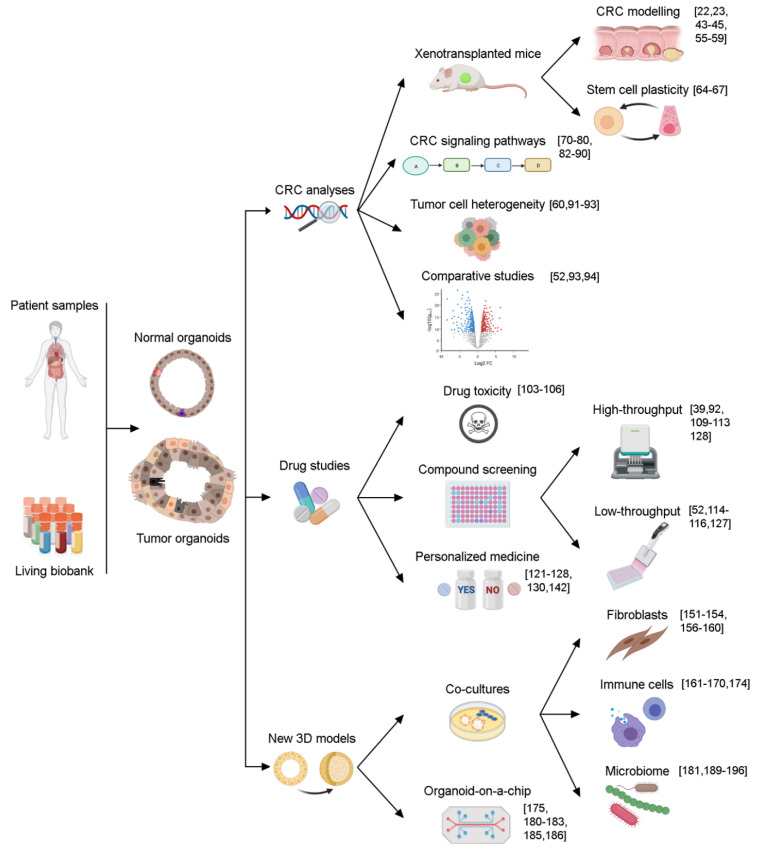
Schematic representation of the uses of normal and tumor organoids for CRC basic and clinical research. References of the original studies are shown in square brackets. Created with Biorender.com (accessed on 27 April 2021).

Organoid technology allows the long-term expansion of normal and tumor tissues without genetic modifications. Initially described in gastrointestinal organs, organoids from non-transformed tissues show an unusual genetic [46,47,48] and epigenetic [49] stability when compared with cell lines, despite extensive serial passaging. Preserved genetic diversity and morphological stability has been observed in organoids derived from CRC primary tumors and their metastases [26,41,50,51,52]. This genetic stability (at least six months in culture) has been found in organoids derived from microsatellite stable (MSS) CRC but not, as expected, in those from microsatellite instable (MSI) CRC [26]. Further advantages of organoids include the possibility of biobanking, studies of cell heterogeneity, transplantation in experimental animals, individual patient analysis, and gene editing. In fact, organoids are a good system to work with clustered regularly interspaced short palindromic repeats (CRISPR)-CRISPR-associated protein 9 (CRISPR-Cas9) technology. These properties make organoids an excellent, unique tool for analyzing gene function and, in combination with CRISPR-Cas9 and orthotopic xenotransplantation in mice, they have emerged as an excellent tool for modelling diseases and maybe for regenerative medicine in the future [53,54]. Figure 2 summarizes the types of CRC studies performed using organoids as a model.

### 3.1. Organoids for CRC Modelling and Cell Plasticity Analyses

As mentioned before, sporadic CRC arises from the serial acquisition of mutations in genes encoding components of relevant signaling pathways such as WNT/β-catenin, RAS-MAPK, PI3K, p53 and TGF-β. However, the lack of systems derived from human normal intestinal tissues made it impossible to develop a good in vitro model that reflected the initiation and progression of human CRC as postulated by B. Vogelstein in the 1990s. The sequential introduction of CRC driver gene alterations affecting the aforementioned signaling pathways (oncogenic mutations in *KRAS* and *PIK3CA* and disruption of *APC*, *TP53* and *SMAD4* tumor suppressor genes) into normal human organoids using CRISPR-Cas9 allowed the modelling of the adenoma-carcinoma progression upon transplantation into mice [43,44,55]. The accumulation of these mutations made normal organoids progress in the absence of essential niche factors and develop tumors after transplantation in immunodeficient mice [43,44]. However, these studies differed slightly about metastatic capability. While Fumagalli and colleagues observed liver metastases after orthotopic transplantation of these engineered-modified organoids [55], Matano and colleagues described only micrometastases after splenic injection. This suggests a possible influence of the microenvironment in the metastatic capacity [43]. A clear result from these studies was that the combined loss of *APC* and *TP53* is sufficient for the appearance of aneuploidy [43]. Furthermore, the progression of serrated adenomas (rare colorectal polyps) has recently been investigated by introducing into organoids chromosome alterations that involve R-spondin (*RSPO*) genes [56].

Genome editing approaches have been performed in mouse organoids to characterize CRC-associated genes and understand tumor progression and metastasis [57,58]. In an advanced CRC model, Batlle’s group crossed mice carrying in intestinal SC different driver CRC gene mutations, generated tumor-derived organoids from the resultant mice, and finally implanted these organoids in normal wild-type animals with the same genetic background. This elegant approach allowed an immunocompetent mouse model of metastatic CRC to be obtained that led the authors to demonstrate the key role that TGF-β plays in the evasion of the immune system [59].

In recent years, the organoid system has become a suitable model to study in vivo tumor evolution and CSC plasticity by combining gene editing and xenotransplantation in mice. Cortina and colleagues used CRISPR-Cas9 to introduce *EGFP* and lineage-tracing cassettes into the *LGR5* locus in PDTOs. The authors found that *LGR5-EGFP* CSC xenotransplanted in immunodeficient mice adopted a hierarchical organization that resembled that of normal colonic epithelium [60]. Many types of intestinal cells at intermediate or even terminal differentiation stages can dedifferentiate following a lethal injury in normal tissues to replenish the SC repertoire, or as a consequence of the acquisition of genetic alterations and/or in response to signals from the tumor microenvironment in cancer. Thus, stemness is today considered a modifiable cellular state instead of a permanent feature of a fixed group of normal or cancer cells [61,62,63]. Two simultaneous studies using CRISPR-Cas9 to label (with GFP) and then eliminate *LGR5^+^* CSC (by means of knock-in Caspase 9 or diphtheria toxin receptor cassettes) smartly demonstrated the plasticity of human and mouse CRC CSC [64,65]. Transplanted CRISPR-engineered organoids in mice showed limited tumor progression or even regression after the ablation of *LGR5^+^* cells. However, after the *LGR5*–specific ablation was turned off, new *LGR5^+^* cells derived from differentiated lineages appeared in both studies. De Sousa e Melo and colleagues did not observe the reappearance of *LGR5-GFP^+^* cells in spontaneous liver metastases after CSC ablation. This suggests that tumor plasticity is niche-dependent [64]. In contrast, a recent report by Fumagalli and colleagues reported that the majority of disseminating mouse CRC cells that seed liver metastases are *Lgr5^−^,* while metastatic outgrowth depends on the reappearance of *Lgr5*. They concluded that this plasticity occurs independently of microenvironmental factors and that *Lgr5^+^* cells are essential for the growth, but not the establishment, of metastases. Therefore, this study suggests that specifically targeting CSC may not be enough to completely prevent metastatic disease [45].

Lentiviral transduction has been used as an alternative strategy to CRISPR-Cas9 for organoid engineering and to study normal and tumor intestinal stem cells [66]. Plasticity during CRC progression was also demonstrated by labelling *ASCL2* (a master regulator of *LGR5*) activity using lentiviruses (a technique called STAR, SC ASCL2 reporter) in human colon organoids [67].

### 3.2. Organoids for Molecular and Genetic CRC Studies

Human and mouse colon organoids have replaced colon carcinoma cell lines as a system to study the role of genes, pathways, compounds and agents in CRC. Organoids have been extensively used to study the WNT/β-catenin pathway, which is essential for normal SC homeostasis at the intestinal crypt bottom and crucial for CRC initiation and progression when deregulated [5,22,68,69]. WNT/β-catenin pathway is abnormally activated in most CRCs either intracellularly (by mutation of *APC*, *CTNNB1* or *AXIN*) or at plasma membrane receptor level (by excessive WNT/R-spondin signaling due to high level of ligands, reduced expression of WNT inhibitors or mutation of *ZNRF3*/*RNF43*). Remarkably, different molecular signatures were found in isogenic human CRC PDOs cultured in the presence or absence of ligands or following CRISPR-Cas9-induced *APC* loss [70]. Interestingly, the authors found that the oncogenic WNT signature (induced by *APC* loss) was associated with good prognosis in CRC while ligand-mediated signaling was linked to poor prognosis [70].

Several recent studies using intestinal organoids deal with the ubiquitin ligases ZNRF3 and RNF43 that act on the WNT receptors Frizzled and LRP5/6, promoting their degradation and thus the inhibition of WNT signaling. Spit and colleagues described novel *RNF43* truncating mutations that do not affect WNT receptors’ turnover but induce β-catenin-mediated transcription, and cooperate with *TP53* loss to drive niche-independent cell self-renewal and proliferation [71]. Another study has shown that RNF43 activity requires its phosphorylation in specific serine residues. When this phosphorylation is abrogated by mutations, the Rnf43 inhibitory action on Wnt signaling in mouse organoids is abrogated [72]. ZNRF3 and RNF43 are themselves regulated by ubiquitination, and USP42 has recently been identified as a deubiquitinase that binds ZNRF3 and stabilizes R-spondin-LGR4-ZNRF3 ternary complex at the plasma membrane. Consequently, USP42 increases the turnover of Frizzled and LRP6 and diminishes WNT signaling in CRC cells and mouse intestinal organoids [73].

Using mouse organoids from three intestinal regions, Wnt signaling has been found to have a variable effect on gene expression and downregulate differentiation genes in a region-specific fashion [74]. Gan and colleagues have reported that protein tyrosine phosphatase receptor type F (PTPRF) interacts with the WNT coreceptor LRP6 mediating the activation of the WNT/β-catenin pathway [75]. In contrast, α-ketoglutarate attenuates WNT signaling and promotes cell differentiation in human PDTOs, which establishes a link between the metabolic microenvironment and CRC [76]. Organoids have also been a useful tool to identify new WNT target genes such as *LARGE2* encoding xylosyl- and glucuronyltransferase 2, which mediates functional O-glycosylation of α-dystroglycan, and so CRC cell adhesion to the ECM component laminin [77].

Molecular and genetics studies using intestinal organoids have also been focused on RasGRP1, which acts as a tumor suppressor in the context of aberrant WNT signaling by inhibiting EGF-driven proliferation [78], and on YAP, a transcriptional activator of the Hippo pathway that when overexpressed has tumor suppressor activity by downregulating Wnt and *Lgr5^+^* cell reprograming [79]. Interestingly, in mouse intestinal cultures the Wnt-regulated CD44 isoform CD44v4-10 promotes the expansion of adenoma organoids induced by hepatocyte growth factor through its tyrosine kinase receptor c-MET [80,81]. PDOs were also used to describe that non-canonical PTCH1/SHH-dependent, GLI-independent Hedgehog signaling is a positive regulator of WNT activation required to block differentiation and increase survival of CRC CSC [82]. In a recent study, Ponsioen and colleagues have analyzed the interplay between EGF signaling and the activation of the MAPK ERK using PDOs from *KRAS* and *BRAF* mutant CRCs. The authors show that without EGFR activity, signaling by these two oncogenes is insufficient to sustain full cell proliferative potential. Thus, both in CRC PDOs and in vivo EGFR activity amplifies the oncogenic MAPK signaling pathway [83].

Human intestinal PDOs and PDTOs, genetically modified by means of CRISPR-Cas9, have been used to study the role of genes and factors in CRC progression [84] and that of genes involved in the resistance to TGF-β-mediated growth restriction [85], as well as to identify tumor suppressor genes in vivo [86]. They have also been useful to address epigenetic studies [87] and to study the origin of mutational signatures after the deletion of DNA mismatch repair genes (*MHL1* and *NTHL1*) [88]. Interestingly, in a series of studies, Espinosa’s group has reported the effect of the deficiency of IKKα and IκBα, two regulators of NFκB signaling, in mouse intestinal organoids [89,90].

The study of intratumor cell heterogeneity is usually associated with the problem of limited biopsy material and hinders in-depth readings in single-cell RNA-sequencing (scRNA-seq) analyses [91]. Roerink and colleagues efficiently overcame this limitation by performing RNA-seq, and mutational and DNA methylation assays with clonal organoids derived from normal and neoplastic colorectal SC isolated from normal crypts or from separate tumor pieces of the same resection specimen. Phylogenetic trees revealed that different clonal organoids derived from the same tumor piece share similarities but also show genetic diversity [92]. Cellular heterogeneity of metastatic CRC has been recently studied by bulk transcriptomic and scRNA-seq analyses of primary and metastatic PDOs. The authors found that metastatic PDOs show decreased expression of differentiated markers and, curiously, also of intestinal stem cell markers, demonstrating that primary and metastatic lesions have distinct cellular composition [93].

Our group has used organoid technology to identify possible differences between colon and rectum tumors. We compared by RNA-seq assays the transcriptomic profiles of normal and tumor PDOs from colon (sigmoid) and rectum [94]. We observed that organoids from both origins shared a similar gene expression profile. However, a group of genes of the biosynthetic machinery were found as rectal tumor organoid-specific, including those encoding the RNA polymerase II subunits POLR2H and POLR2J. This agrees with the clinical differences between colon and rectal cancers [94].

## 4. Organoids as a Platform for Screening Assays

For decades, most compounds that showed cytotoxic activity in the classical test panel of cultured cancer cell lines failed in the early phases of clinical studies. This low success rate is attributed to the dissimilarity between patient tumors and genetically unstable immortal cell lines after prolonged 2D culture on plastic [95]. As discussed by Fujii and Sato, the disadvantages of cancer cell lines include that they do not represent the whole tumor and that most of them derive from high-grade tumors. Consequently, the tumors that they generate when injected into animals are poorly differentiated and do not illustrate the diversity of human cancers [3]. PDTOs show advantages over cancer cell lines: they can maintain genetic stability over the long-term in culture, retain cell−cell and cell−matrix interaction, and recapitulate the features of the original tumors from which they are derived (such as the histology and mutational landscape). In terms of drug sensitivity, they may provide personalized data of clinical utility. PDTOs can be generated from a variety of tumors, are easier to establish and reflect tumor heterogeneity (Table 2).

Genetically-engineered mouse models and patient-derived xenografts (PDX) generated in mice were important advances in mimicking tumor niche, although they have considerable limitations. Compared to PDX, PDTOs save animal testing, have a lower cost:benefit ratio, require less specialized facilities, can be stored as living biobanks, and are more amenable to drug screening [96,97] (Table 2). Organoids are therefore a suitable complement to tumor genome sequencing and xenograft studies for predicting patient responses to treatments and could in the future be responsible for the reduction of animal testing in biomedical research [2,98,99,100,101].

Over the last few years, many groups have demonstrated the advantages of using PDOs for drug screening assays, specifically in three contexts: (i) toxicity studies, (ii) drug discovery, and (iii) precision medicine.

### 4.1. Toxicity Studies

Failures in many clinical trials are mostly related to problems with efficacy or harmful side effects. Drug toxicity has usually been analyzed using xenografted immunocompromised mice with limited translatability to the clinic. To overcome these problems, biomarkers of damage in normal tissue (traditionally considered liver, kidney and the vascular system) must be identified [102]. In this context, organoid technology has emerged as a unique system to test drug effects in normal and tumor cells from the same patient. Moreover, it allows the study of the in vivo effect of a drug in mice and subsequent generation of normal and tumor organoids to analyze their genetic and transcriptomic profiles.

Lu and colleagues successfully used mouse small intestine organoid cultures to study drug metabolism and toxicity [103]. Several recent reports using intestinal organoids showed the adverse consequences of treatment with 5-fluorouracil (5-FU), a drug commonly used to treat CRC patients. Genome-wide analysis performed in isogenic clonal normal organoids from human small intestine and in tumor biopsies from metastatic CRC patients revealed the apparition of a mutation signature after 5-FU treatment in both systems [104]. Moreover, Cho and colleagues have shown in CRC PDTOs that 5-FU induces the activation of CSC via the WNT/β-catenin pathway. This suggests that a combined treatment of 5-FU with WNT inhibitors would be an efficient strategy to avoid tumor relapses [105]. In addition, Engel and colleagues examined the expression of stem cell markers in a cohort of CRC PDTOs and their correlation with sensitivity to 5-FU treatment. They found that clusterin expression was significantly enriched in PDTOs treated with 5-FU and correlated with the level of drug resistance. Importantly, clusterin expression was associated with lower patient survival and an increase in disease recurrence. This suggests that clusterin is a marker of drug resistance and may identify cells that drive CRC progression [106].

### 4.2. Drug Discovery

The use of organoids for anticancer drug discovery has recently been extensively reviewed by Rae and colleagues [107]. A brief historical summary regarding CRC must start with Ashley and colleagues, who were the first to perform a proof-of-concept study using primary cultures of CRC cells for preclinical drug testing [108]. Subsequently, some groups demonstrated the validity of CRC PDOs for testing many anticancer drugs using robotized procedures [41,92,109]. Thus, van de Wetering and colleagues reported the lack of response to EGFR inhibitors of organoids with mutated *KRAS* and that nutlin was only active in wild-type *TP53* organoids [41]. Other groups used their CRC organoid biobanks to perform high-throughput analyses to identify potential inhibitors of *KRAS* and *EGFR* oncogenes [110,111] and to study the regenerative response of normal intestinal organoids to nearly three thousand compounds [112]. Recently, Huelsken´s group has developed a high-content high-throughput screening system in small intestine mouse organoids to identify small-molecule drugs that are able to induce the differentiation of intestinal wild-type and cancer cells [113]. Although assay variability was a problem in early studies [41], this issue can be overcome at least at a low-scale by selecting the number and size of CRC PDTOs by an individual pre-analysis [114].

Organoid technology is also a unique system to elucidate the molecular and functional role of diverse agents in human disease in epithelial cells [115,116]. Based on the antitumor action of the active vitamin D metabolite 1α,25-dihydroxyvitamin D_3_ (calcitriol) in CRC and other carcinomas and on its multilevel antagonism of the WNT/β-catenin pathway [117], our group has investigated the effects of this hormonal agent on human normal and tumor PDOs. RNA-seq assays revealed the profound and distinct regulatory effects of calcitriol on the transcription pattern in both types of PDOs [52]. In normal PDOs, calcitriol induced stemness-related genes (*LGR5*, *SMOC2*, *LRIG1*, *MSI1*, *PTK7* and *MEX3A*) and reduced cell proliferation without affecting WNT target genes. Supporting this, chromatin immunoprecipitation-seq assays identified several stemness genes (*SMOC2*, *MSI1*) as direct targets of calcitriol in PDOs. By contrast, in PDTOs, calcitriol induced cell differentiation traits and variably diminished proliferation, with only minor effects on stemness genes [52]. Thus, vitamin D appears to have a homeostatic action on normal human colon SC and an antitumor prodifferentiation action on CRC CSC.

### 4.3. Organoids for Personalized Treatment of CRC Patients. Early-Onset CRC and Colorectal Carcinomatosis

Precision medicine claims to customize therapies by identifying the most suitable treatment based on genetic, transcriptomic and functional information derived from each patient. Organoids are the only system that allows the long-term culture of patient SC in vitro, and makes it possible to replicate ex vivo primary cancers, infectious or developmental diseases directly from a biopsy of the patient. Cystic fibrosis (CF) was the first disease to be addressed by the organoid approach. The Clevers’ group generated rectal organoids directly from CF patients and analyzed their functional response to available therapeutic agents in vitro, identifying potential target patients for the drugs [118,119].

Organoids can be used to predict clinical outcomes in CRC [120]. In a first study, the comparison of the therapy response of 23 colorectal and gastroesophageal cancer patients in clinical trials and that of their PDTOs showed an 88% positive predictive value and, importantly, a 100% negative predictive value [121]. More recently, precision medicine studies have been extended to several types of cancer and treatments, demonstrating the feasibility of PDOs for research and treatment testing, and as a promising tool for predicting patient responses [1,122,123,124,125,126,127,128]. Colonic organoids derived from human iPSCs have also been used for CRC drug testing [129]. Thus, organoids have the potential to reduce inefficient medication, deleterious side-effects, and health care costs. The first formal, prospective intervention trial, the Selecting Cancer Patients for Treatment Using Tumor Organoids (SENSOR) trial, was designated to evaluate the potential and feasibility of using PDTOs to guide experimental treatment decision in metastatic CRC patients without curative treatment options. The results have not demonstrated concordance between drug sensitivity of organoids and patient clinical responses [130]. As said by the authors, putative reasons for this include the low culture success rate (due to the small size and low quality/small amount of tumor cell content of biopsies), clinical deterioration of patients during standard of care and the insufficient rational design of drug panels.

PDTOs can be useful to clarify the conflicting literature on the predictive importance of primary tumor location on anti-EGFR antibody therapy in metastatic CRC patients with wild-type *KRAS*. Thus, some studies have found a greater effect of chemotherapy plus anti-EGFR as compared to chemotherapy alone or chemotherapy plus anti-VEGFR in patients with left-sided tumors, but not in patients with right-sided tumors [131,132,133]. However, tumor sidedness was not found to be associated with response to treatment in other studies [134]. In this scenario, the analysis of the response to individual and combined treatments with these agents of PDTOs generated from either left or right colon tumors may contribute to solve the controversy. 

The increasing incidence of CRC in Western countries in the population under 50 years of age (early-onset CRC, EOCRC) contrasts with the reduction in those over this age and makes EOCRC a highly interesting subject of study. There are two types of EOCRC: familial, due to germline mutations like those causing familial adenomatous polyposis or hereditary non-polyposis colorectal cancer, or sporadic. The reasons (genetic, epigenetic, dietary, lifestyle, etc.) for the increased rates in sporadic EOCRC rates are unknown. A first organoid biobank enriched in EOCRC has been recently reported [135]. Exome and transcriptomic analyses have revealed a diversity of molecular phenotypes, including some unusual alterations such as *PTPRK-RSPO3* fusions whose relevance must wait for new and larger studies.

Peritoneal metastases (carcinomatosis) are a progression feature of a subset of colorectal, gastric and ovarian cancers. Colorectal carcinomatosis have poor prognosis and limited treatment options. The use of organoids to study peritoneal metastases is far behind that of other cancer systems. Only a few studies have recently been reported, usually involving very few patients and/or organoids that were generated following transplantation of human biopsies in mice [136,137,138]. Furthermore, in some cases the so-called organoids were in fact short-lasting primary heterogenous cultures from ovarian or gastric ascites-derived metastases [139,140,141]. No consensus culture media or biobanks have existed up to now. The most advanced study is probably that reported by Narasimhan and colleagues, who have been able to generate and validate 19 organoids from colorectal peritoneal metastases of 28 (68% success rate) patients undergoing standard care [142]. Drug profiling within eight weeks after surgery allowed the ranking of sensitivities that led to a treatment change for two patients. This study shows the feasibility of the organoid approach to guide therapeutic choices. Clearly, peritoneal metastases organoids are an unresolved issue that warrants effort to improve patient survival.

## 5. Organoid Limitations and New 3D Models

Organoids have many advantages that suggest they are a unique tool for studying the biology of normal and tumor tissue. However, they also show some limitations. Advantages and limitations have recently been reviewed by Rae and colleagues [107]. In addition to intrinsic experimental difficulties, such as the frequent small size of tissue samples (particularly if they are diagnostic endoscopy biopsies) and bacterial contamination, the success rate for PDTOs is affected by the characteristics of the tumor (mutational status, subtype) and the difficulty to predict an adequate culture medium for each tumor.

Intratumor heterogeneity is another issue to take into account. During the first stages after establishment, PDTOs show substantial genetic stability and genetic heterogeneity. However, maintenance of this situation over the course of the culture period cannot be predicted. Furthermore, tumors are dynamic entities that change over time in therapy-naive patients, especially in MSI CRC, and particularly following chemo- or radiotherapy. Thus, organoids established at one timepoint only faithfully represent the tumor at the precise developmental time of their establishment. As stated by Fujii and colleagues, generating clonal organoids from single cells at culture initiation or at early passages may be highly relevant for specific purposes [26].

To circumvent the animal origin of ECM, many natural and synthetic hydrogels are in development to substitute Matrigel and BME. However, they need to satisfactorily overcome several issues: adequate binding of cells, a specific composition that resembles the niche conditions and the induction of physiological signaling. Other open issues for Matrigel, BME and hydrogels are cytotoxicity, optimal stiffness, and batch variability [143,144,145,146].

Importantly, a main limitation of ASC-PDOs is that the structures are made exclusively of epithelial cells and lack the stroma: fibroblasts, immune cells, nerves and vessels. The stroma or tumor microenvironment (TME) is largely responsible for tumor heterogeneity and plasticity and may contribute to CRC progression by means of reciprocal interactions with carcinoma cells [147]. Now, heterotypic structures that are more complex than PDOs rely on the transient co-culture of PDOs with stromal cell types that increase cell−cell interactions, or the use of short-lived tissue or tumor explants, which cannot be considered PDOs [148,149,150]. Therefore, many studies are trying to develop a new generation of heterotypic PDOs via co-culture with non-epithelial stromal cells such as cancer-associated fibroblasts (CAF), enteric nervous system, endothelial and immune cells. The ECM responsible for providing structural and biochemical support to organoid epithelial cells has made it easier to generate co-culture models with diverse TME cell types. However, a challenge for these studies is the media requirements of each cell type.

### 5.1. Co-Culture with Fibroblasts

Some reports have successfully achieved co-culture systems based on human colon organoids with fibroblasts and nervous system cells [151]. Mouse organoids have also been co-cultured with mouse embryonic fibroblasts (MEF) [152]. This study describes the generation of small intestine organoids from epithelial-specific porcupine-deficient mice. Although Wnt-producing epithelial cells of these organoids cannot secrete Wnt factors due to the lack of porcupine, this function is replaced by MEF, showing that stromal Wnts are sufficient to maintain small intestine SC. Other studies describe that the co-culture of intestinal crypts in the presence of myofibroblasts or their conditioned medium increases the efficiency of organoid formation from isolated crypts [153,154]. A recent study used scRNA-seq and high-resolution microscopy to identify the mouse intestinal stromal cells responsible for the secretion of BMP factors and their inhibitors and employed co-cultures of these cells with intestinal crypts to study the effect of the physiological BMP gradient [155,156].

Interestingly, fibroblast-derived extracellular vesicles induce colony formation of CRC organoid cells in hypoxia, which potentially contributes to tumorigenesis under unfavorable conditions in CRC by transmitting WNT and EGF [157,158]. Mosa and colleagues reported that Wnt activity in CAFs is linked to distinct CAF subtypes: low or high Wnt levels induce inflammatory-like CAFs (iCAFs) or contractile myofibroblastic CAFs (myCAFs) subtypes, respectively. These authors showed that the co-culture of tumor organoids with iCAFs resulted in a significant upregulation of epithelial-to-mesenchymal markers, while myCAFs reverted this phenotype [159]. The co-culture of mouse intestinal organoids with macrophages and fibroblasts also revealed that these stromal cells can hyperactivate the PI3K signaling in colonic epithelial cells that already carry *Kras* and *Tp53* mutations and that oncogenic mutations cell-autonomously mimic an epithelial signaling state normally induced by stromal cells [160].

### 5.2. Co-Culture with Immune Cells

PDOs allow the co-culture of primary normal or tumor intestinal epithelium with immune cells to investigate their cross-talk and relevant aspects of the defense against infections and immuno-oncology. A first co-culture of human primary monocyte-derived macrophages and small intestine 3D organoids converted into 2D monolayers on permeable inserts was generated to evaluate barrier function and cytokine secretion under basal conditions and following bacterial infection [161]. The activation of the peripheral T-cells acquiring features (morphology, markers, motility) of intraepithelial lymphocytes has also been reported when they were co-cultured with intestinal organoids [162,163]. The air−liquid interface primary tumors en bloc and tumor infiltrating lymphocytes were used to study the cytotoxicity of the immune checkpoint blockade anti-PD-1 and anti-PD-L1 antibodies [164]. The profiling of PD-1 blockade has also been analyzed in short-term 3D microfluidic cultures of patient-derived organotypic spheroids retaining autologous lymphoid and myeloid cell populations [165]. Limitations to these studies are the restriction to pre-existing tumor infiltrating immune cells or peripheral lymphocytes and the preservation of their activity and viability in culture.

Importantly, a recent study has demonstrated that MSI CRC and non-small cell lung cancer organoids can be used to assess the efficiency of T-cell-mediated tumor killing. Thus, the co-culture of peripheral T-cells and these PDTOs induces the generation of tumor-specific T-cells that do not recognize normal organoids or tissue [166]. This system allows the expansion of reactive tumor-specific T-cells for personalized analysis of their anticancer properties, offering the possibility of comparing the relative contribution of different cancer antigens to T-cell killing. In addition, it allows the assessment of how the genetic state of the tumor cells influences sensitivity to T-cell attack, and the evaluation of strategies of adoptive T-cell transfer. The procedure consists of an initial expansion during two weeks of tumor-reactive T-cells in co-cultures of peripheral blood lymphocytes and PDTOs in the presence of γ-interferon. Then, the effector functions of T-cells after the recognition of tumor cells and their capacity to kill PDTOs are evaluated [167]. Interestingly, CRC organoids have also been used to develop a platform to study chimeric antigen receptor (CAR)-engineered lymphocytes in a personalized manner [168]. Thus, PDTO-immune cell co-cultures may be useful in several types of anticancer therapies and in immunological research in general [169]. Human and mouse colon organoids have recently been used to study interleukin regulation of innate immune signaling [170].

Inflammatory bowel diseases (IBD: Crohn’s disease and ulcerative colitis) are chronic inflammatory disorders affecting different gut segments with genetic and environmental (infections, etc.) etiology that in the long-term increase the risk of developing CRC. To obtain further insight into the genetics and biology of these disorders, organoids have been established from IBD patients [171,172,173]. In this context, the effect of blood human type 1 T-regulatory (Treg) cell supernatants has been investigated in colon organoids from patients with Crohn’s disease or ulcerative colitis [174].

### 5.3. Organoid-on-a-Chip 

The usual cystic nature of organoids with the lumen oriented towards their interior is an important limitation since it restricts their size and useful life. It also prevents the study of the response to signals, microbiome and drugs acting on the apical surface and elimination of cell debris and dead cells and so, an adequate homeostasis. A further sophistication of organoids aimed at recreating a more physiological environment uses bio-engineered devices or scaffolds [175,176]. 

Organs-on-a-chip have been defined as “microfluidic cell culture devices, originally fabricated using methods adapted from computer microchip manufacturing, which contain continuously perfused chambers inhabited by living cells arranged to simulate tissue- and organ-level physiology” [177]. The combined use of organoid technology and organ-on-a-chip engineering constituted a step ahead in the development of 3D culture models and led to the creation of the so-called organoids-on-a-chip [178,179]. Currently, the possible synergy between these two techniques is an open issue and organoids-on-a-chip from different tissues have already been published. Focusing on the intestine, Inger’s group was ground-breaking using this technology in the gut by generating an organoid-on-a-chip, using PDOs from the small intestine [175] and later from the colon [180]. Nikolaev and colleagues also developed a system that combines tissue engineering using a perfusable tube scaffold (which is used as a guide) with the self-organizing capacity of mouse small intestine organoids [181]. 

This breakthrough has given rise to several possibilities for customizing microfluidic devices such as co-cultures of organoids with stromal cells. Thus, Verhulsel and colleagues developed a 3D biomimetic scaffold made entirely of collagen treated with threose that cross-links the collagen and thus maintains the desired topography allowing the co-culture of fibroblasts and intestinal organoids [182]. Colon organoid co-cultures within a self-assembled vascular network have recently been successfully achieved, leading to better cell/organoid growth under constant perfusion than with a conventional static condition [183]. These organoids-on-a-chip have also been proposed as platforms for testing drug activity and toxicity [184,185,186]. 

## 6. Organoids to Study the Intestinal Microbiome and Host-Pathogen Interactions

The intestinal microbiome or microbiota is a dynamic variety of microorganisms (bacteria, viruses, and fungi) that play a key role in gut homeostasis and protection against pathogen infection. Many studies have proposed a link between the intestinal microbiome and CRC. Supporting this, CRC patients have altered gut microbial composition and diversity, which can contribute to CRC tumorigenesis by the secretion of carcinogenic metabolites and/or the alteration of the host metabolism and immune system [187,188].

PDOs have been described as a model to study host−pathogen interaction, including the role of the microbiome in CRC [3]. Several protocols were used: microinjection of microbiome or their metabolites into the organoid lumen, dissociation of organoids and reseeding onto Transwell inserts on Matrigel-coated dishes followed by exposure of the apical/luminal surface to microbes or their metabolites added to the culture medium, and disruption of organoids into suspensions that are mixed with microbes before cultivation in 3D matrices to reform organoids [189,190,191]. Co-cultures with microorganisms have also been performed in organoid-on-a-chip devices [181,192]. Remarkably, a recent study by the Clevers’ group has demonstrated a direct role of the genotoxic *pks^+^* strain of *E. coli* in CRC tumorigenesis. This bacterial strain is more frequently present in CRC patients than in healthy individuals and synthesizes colibactin, a compound that induces double-strand DNA breaks in cultured cells. The authors found that after repeated injections of *pks^+^ E. coli* into intestinal PDOs, they acquired a mutational signature that coincided with that detected in a subset of human CRC genomes [193].

Intestinal organoids are also emerging as a model system to investigate host−parasite and host−virus interactions [3,194]. Among the latter, infection by several enteroviruses and coronaviruses have been investigated including SARS-CoV-2 [195,196].

## 7. Perspectives and Conclusions

CRC is a major health problem worldwide and the absence of a good ex vivo system has for a long time been a hurdle to improve patient management in the clinic. Organoid technology has emerged as a new strategy to tackle this disease. In spite of several drawbacks and limitations, PDOs are today considered the preferential model to study CRC. In combination with cutting-edge techniques such as CRISPR, scRNA-seq and orthotopic xenotransplantation, organoids may significantly contribute to CRC modelling and knowledge.

Organoids have raised great expectations to improve important issues such as the screening of antitumoral drug activity and the toxicity/therapeutic window, and the personalized sensitivity to chemotherapy and radiotherapy. However, the still low number of studies completed and reported reveals that substantial problems remain unsolved. Two of these are the reproducibility of assays, which, at least in part, is due to the absence of standardization of culture media and conditions, and the intrinsic variability and suboptimal features of patient biopsies and cohorts. The latter includes the frequent small size and elevated cell heterogeneity of samples and the differences between therapy-naïve patients and those who have been subjected to a variable number of distinct types of therapies.

Hopefully, improvements in the field should come from the optimization of organoids with the incorporation of stromal components and the generation of organoid-on-a-chip devices. These systems will constitute models closer to the in vivo situation where treatments, not only against tumor cells but also directed to stromal cells, can be tested potentially in a dynamic fashion, involving multiple intercellular interactions.

Clearly, the definitive utility of PDTOs in personalized medicine will result from the accumulation of studies investigating their predictive value. Available data and ongoing studies point towards the desired precision treatments that will hopefully contribute to improving the outcome of CRC patients.

## Figures and Tables

**Table 1:** Composition of growth medium for human and mouse organoid cultures.

Reagent	Function	Human		Mouse
		Normal	Tumor	Normal
Advanced DMEM/F12	Basal medium	+	+	+
HEPES	pH buffer	+	+	+
Glutamax	L-glutamine supplier	+	+	+
B27 supplement	Vitamins/cofactors mix	+	+	+
N2 supplement *	Vitamins/cofactors mix	+/−	+/−	−
N-acetyl-L-cysteine	Antioxidant	+	+	+
Antibiotics	To avoid bacterial contamination	+	+	+
Wnt3A-cond. medium	Stemness/Wnt signaling activator	+	+ ^#^/−	+
RSPO	Stemness/Wnt signaling enhancer	+	+ ^#^/−	+
Nicotinamide *	Stemness/cystic phenotype	+/−	+/−	−
PGE_2_ *	Wnt activator/cystic phenotype	+/−	+/−	−
EGF	Mitogen	+	+	+
Gastrin	Mitogen	+	+	−
FGF-2	Mitogen	+ ^¥^/−	+ ^¥^/−	−
IGF-1	Mitogen	+ ^¥^/−	+ ^¥^/−	−
Noggin	BMP inhibitor	+	+	+
Y-27632 ^†^	ROCK/*anoikis* inhibitor	+	+	+
SB202190	p38MAPK inhibitor	+/− ^¥^	+ ^#^/− ^¥^	−
A 83-01/LY2157299	TGF-β inhibitor	+	+	−

+, Required; −, Absent; * Not strictly required [25]; ^#^ For certain tumor types [26]; ^†^ Only at single-cell stages; ^¥^ Published by Sato’s group [24,27].

**Table 2:** Comparison among patient-derived colorectal cancer model systems.

Features	Cell Lines	PDX	PDTO
Initiation success rate	Low	Moderate	High
Possibility of expansion	Very high	Moderate	High
Time consuming	Low	High	Moderate
Cost	Low	Very high	High
Expertise	Low	High	High
Ease of maintenance	High	Moderate	Moderate
Amenability for genetic manipulation	High	Low	High
Ease of downstream assays	High	Low	High
High-throughput drug screening	Very high	Low	High
Low-throughput drug screening	Very high	Moderate	Very high
Modelling early stage CRC	No	No	Yes
Autologous normal controls	No	No	Yes
Isogenic control by genome editing	Yes	No	Yes
Patient-specific	No	Yes	Yes
Save animal testing	Yes	No	Yes
Recapitulation of tissue features	Low	High	High
Need of specialized facilities	Low	High	Moderate
Cell-matrix interaction	No	Yes	Yes
Lack of microenvironment	Yes	No *	Yes

* Non-human microenvironment.
